# Features of hardware implementation of quasi-continuous observation devices with discrete receivers

**DOI:** 10.1186/s42492-022-00102-7

**Published:** 2022-02-08

**Authors:** Oleksandr Maryliv, Mykhailo Slonov

**Affiliations:** Military-Diplomatic Academy named after Eugene Bereznyak, Kyiv, 04050 Ukraine

**Keywords:** Discrete receivers, Formalization of tasks, Conditions of hardware implementation

## Abstract

This article proposes an approach to the formalization of tasks and conditions for the hardware implementation of quasi-continuous observation devices with discrete receivers in remote sensing systems. Observation devices with a matrix are used in medicine, ecology, aerospace photography, and geodesy, among other fields. In the discrete receivers, the sampling of an image in the matrix receiver into pixels leads to a decrease in the spatial information of the object. In a greater extent, these disadvantages can be avoided by using photosensitive matrix with a regularly changing (controlled) density of elementary receivers-matrix (RCDOER-matrix). Currently, there is no substantiation of the tasks and conditions for the hardware implementation of RCDOER-matrix. The algorithmic formation of a quasi-continuous image of observation devices with the RCDOER-matrix is proposed. The algorithm used a formal pixel-by-pixel description of the signals in the image. This algorithm formalizes the requirements for creating a photosensitive RCDOER-matrix of a certain size, as well as for changing the mechanism for forming and saving a frame with observation results. The application of the developed method will allow multiplying the pixel size of the image relative to the pixel size of the RCDOER-matrix. Developed algorithms for RCDOER-matrix are supplemented by formalizing the tasks that arise when creating prototypes. In addition, the conditions for hardware implementation are proposed, which ensure the completeness of registration of the observation picture, and allow avoiding excessive pixel measurements. Thus, the results of the research carried out approximate the practical application of RCDOER-matrix.

## Introduction

Electronic images in observation devices are obtained using the photosensitive matrix as a receiver. Matrix has a pixel size that allows for our eye to receive an image as continuously. Only at high magnification, the image is structured into individual pixels. Each of the pixels is in the state ${\tilde {x}_j}$, which depends on the brightness *u*_*j*_ of corresponding area in observation picture.

With such approach, the appearance of the *U* object can be considered as a set of *N* conjugate sections ${u_j}$ in the space Ω [1]:

1 $$U=U\left\{ {{u_j}:{u_j} \in {\Omega} ,j=\left( {\overline {{1,N}} } \right)} \right\}$$

According to Eq. (1), the image *X* of the object’s appearance is a set of signals ${\tilde {x}_j}$ [2, 3] functionally related to ${u_j}$ from photosensitive matrix:

2 $$X=f\left( U \right)=X\left\{ {{{\tilde {x}}_j}:x \in {\Omega} ,j=\left( {\overline {{1,N}} } \right),{{\tilde {x}}_j}=f\left( {{u_j}} \right)} \right\}$$

Discretizing the image in the receiver into individual pixels leads to the loss of a portion of the spatial information. Within each pixel, there is no information about *x* in ${\tilde {x}_j}$. If the space ${\Delta} {\Omega}_j$ corresponds to an individual pixel, the description of image *X* in Eq. (2) can be refined as follows:

3 $$X=X\left\{ {{{\tilde {x}}_j}:x \in {\Omega} ,j=\left( {\overline {{1,N}} } \right),{{\tilde {x}}_j}=\overline {{x\left( {{\Delta} {{\Omega}_j}} \right)}} \to x \approx {{\tilde {x}}_j}} \right\}$$

Some details of the image of an object fall on the insulating surface Δ in the interim of pixels in matrix. Such details are not involved in photoelectric conversion of the value ${\tilde {x}_j}$.

The photosensitive matrix is designed to minimize losses. The insulating surfaces Δ must be maximally thin, and the pixels maximally small. It can be written as follows:

4 $${\Delta} {{\Omega}_{\text{j}}} \to 0,{\Delta} \to 0,{x_j} \to {\tilde {x}_j}:j=\left( {\overline {{1,N}} } \right)$$

Spatial discretization of the domain Ω in the observation devices depends on the design of the photosensitive receiver. Its standard design contains a system of elementary photosensitive receivers. The rectangular layout system assumes the presence of *m* rows with *n* elementary receivers.

The pixel size of the receiver is equal to the total number of elementary receivers. A signal is generated from each elementary receiver during an exposure. Such a signal sets the state of a specific pixel in the image. With such logic of operation, both the image and the receiver pixel sizes are the same. Then

5 $${\Omega} =\left\{ {{\Delta} {{\Omega}_j}} \right\},j=\left( {\overline {{1,m \times n}} } \right)$$

According to Eq. (5), with a decrease of ${\Delta} {{\Omega}_j}$ the number of receivers increases. In this case, it is necessary to leave the insulating surfaces Δ maximally thin. Equation (4) can be refined as follows:

6 $$m \times n \to \infty ,{\Delta} \to 0,{x_j} \to {\tilde {x}_j}:j=\left( {\overline {{1,N}} } \right)$$

Thus, a successful species observation in advance presupposes the presence of an unstructured receiver. The full implementation of such requirements is technologically and physically difficult. The search of opportunity to fulfill Eq. (6) involves following hardware and algorithmic solutions: (1) constructive improvements [4, 5] in using multi-camera observation devices [6] (which, however, complicate the design of observation devices); (2) a decrease in the size of an elementary receiver to the diffraction limit, so more receivers can be placed on a unit of surface matrix [7, 8] (the implementation of which is technologically complicated and limited by theoretical limits of applicability); (3) consideration of the design of the real tasks of a receiver for observing objects [9] (it reduces the possibility of observing all objects in the image); (4) taking into account the spectral properties of objects of observation and using additional signs of their recognition [10, 11] (during the hyper-spectral observation of an object, a large amount of information is accumulated, and its interpretation requires a high and specific operator qualifications); (5) use of regularly changing (controlled) density of elementary receivers-matrices (RCDOER-matrices) in the structure of the species tool (RCDOER-matrices is only declared, the tasks and conditions of effective work are not formulated); and (6) application of special procedures for processing observation results [12–16] (the possibilities of algorithmic processing are a posterior, and they are always limited to the results of apparatus observation).

As a result, the problem of using RCDOER-matrices in systems of observation has not been completely solved at present.

The aim of this study is to formalize the tasks and conditions for the hardware implementation of quasi-continuous observation devices with discrete receivers in remote sensing systems. This goal is achieved because of the algorithmic formation of the RCDOER-matrix. The algorithm used is a formal pixel-by-pixel description of the signals in the image and the elements of the matrix calculus.

## Methods

The use of the RCDOER-matrix provides element-by-element scanning of the optical image. The photosensitive RCDOER-matrix is characterized by reduced density of distribution of receivers. The density is specified by the multiplicity of their arrangement along rows *k*_1_ and columns *k*_2_. The fold in both directions is estimated by the ratio of the distance between two adjacent receivers to their linear size.

During the scanning, RCDOER-matrix is shifted within the optical image of the object. The nature of the displacement provides a view of all the details of the image. In the initial position and after each displacement by the specified number of pixels, the *X*_*r*_ snapshot is taken. The number of *K* snapshots provides the scanning of all elements of the optical image. Combining all *K* snapshots forms an *X* frame with the object image.

The formalization of the frame of the electronic image *X* will be as follows [14]:

7 $$X=\left\{ {{X_r}} \right\}=\sum\limits_{{r=1}}^{{r=K}} {{X_r}}$$

8 $$ {X_r}={X_r}\left\{ {{{\tilde {x}}_{r,j}}:{x_{r,j}} \in {\Omega} ,j=\left( {1,m \times n} \right),r=\left( {1,K} \right), K=\mathop {\hbox{max} }\limits_{{1 \leqslant r \leqslant K}} r,{{\tilde {x}}_{r,j}} \to {x_{r,j}}} \right\}$$

In Eqs. (7) and (8), the maximum possible reduction in the size of individual pixel is taken into account. Its dimensions are limited only by the physical feasibility. The frame is composed of several snapshots. It allows increasing the pixel size. This is achieved through an intra-matrix scanning of the optical image.

Let us formalize Eq. (7) for reading and memorization in the observation devices. In Eqs. (3) and (8), an image in a frame is considered as collection of individual pixels. This collection is determined by the sign of the summation. The similarity of a digital image to an optical image is achieved because of the systemic arrangement of the elementary receivers of the photosensitive matrix. These are arranged in rows and columns. Thus, the order of reading and storage of digital snapshots of one frame in a matrix form is determined.

In the RCDOER-matrix, *x*_*r,j*_ is a response to the input signal of pixel from *j*-th column and *i*-th row [4]. A matrix **X**_*r*_ of signals from the *r*-th snapshot (*r* = 1, 2, …, *K*) consists of the signals *x*_*r,j*_ of all elementary receivers of the RCDOER-matrix. Equation (7) can be written in a matrix form as follows:

9 $${X_{\text{r}}}=\sum\limits_{{j=1}}^{{j=m}} {\sum\limits_{{i=1}}^{{i=n}} {{x_{i,j}}} }$$

Its dimensions of the matrix **X**_*r*_ are (*m* × *n*). The matrix of **X** is obtained by summing the snapshots *K*. The elements of **X** are signals *x*_*r*,*i*,*j*_:

10 $${X_{\text{r}}}=\sum\limits_{{r=1}}^{{r=K}} {\sum\limits_{{j=1}}^{{j=m}} {\sum\limits_{{i=1}}^{{i=n}} {{x_{i,j}}} } }$$

The dimension of the matrix is then defined as follows:

$$\left( {m \times n} \right) \times K=\left( {m \times n} \right) \times \left( {1+{k_1}} \right) \times \left( {1+{k_2}} \right)$$

**Fig. 1:**
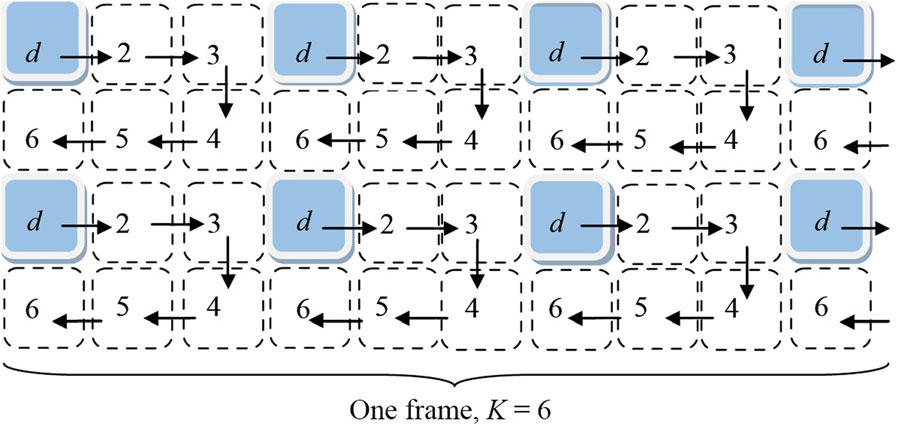
Formation of the frame by the RCDOER-matrix

An example of a frame formation is shown in Fig. 1 [14]. It corresponds to the operation of a RCDOER-matrix with multiplicities of placement *k*_1_ = 2 and *k*_2_ = 1. Pixels *d* are physically present on the matrix. The rest of pixels are virtual.

The frame consists of six snapshots. At the initial moment of time, the first snapshot is exposed. Its image is constructed using signals from elementary receivers *d*. This is snapshot 1  *X*_1_. Next images are exposed after moving the matrix along the trajectory of positions 2, 3, 4, 5, and 6 at a distance *d*. The following pictures are exposed in each of the positions: *X*_2_, …, *X*_6_.

Each snapshot *X*_*r*_ includes pixels (*m* × *n*). Each pixel is a carrier of a signal about the brightness of a point in the environment associated with it. The total number of pixels in *K* frames *m*_Σ_×*n*_Σ_ is equal to the number of discretization elements of the environment. The image has a pixel size *K*-fold larger than the pixel size of the photosensitive matrix.

The hardware implementation of this method involves solving two problems. A photosensitive RCDOER-matrix of size (*m* × *n*) needs to be created with reasonable multiplicity of placement for pixels horizontally and vertically.

The pixel density is set by the distance between adjacent horizontally and vertically pixels. This is a multiple of the size of the pixel size *d*. In such a case, RCDOER-matrix is equivalent to a matrix of size $m_{\Sigma} \times n_{\Sigma}$. This is the virtual pixel size.

In a conventional matrix, the pixel size increases as the surface area increases or the pixel area decreases. In a RCDOER-matrix, an increase in the pixel size is achieved by a decrease in the pixel distribution density. In this case, the multiplicity of the pixel placement increases. The virtual pixel size of the RCDOER-matrix $m_\Sigma \times n_\Sigma$ is calculated as follows:

11 $$ m_\Sigma \times n_\Sigma =\left( {m \times n} \right)\times K=\left( {m \times n}\right)\times\left[ {\left( {1+{k_1}} \right)\times \left( {1+{k_2}} \right)} \right]$$

A regularly changing density of the RCDOER-matrix of the elementary receivers of the same pixel size, but with different multiplicities of placement, will have different virtual pixel sizes. The pixel size of the image is determined by the virtual size of the RCDOER-matrix, which will increase the resolution and quality of the image.

## Results and Discussion

**Fig. 2:**
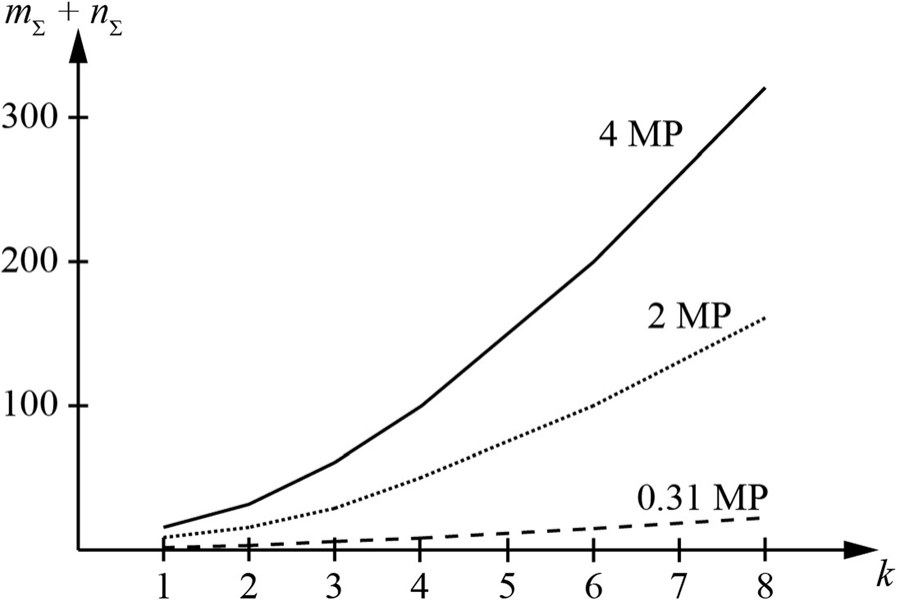
Virtual pixel size of photosensitive RCDOER-matrix with different physical pixel size

**Fig. 3:**
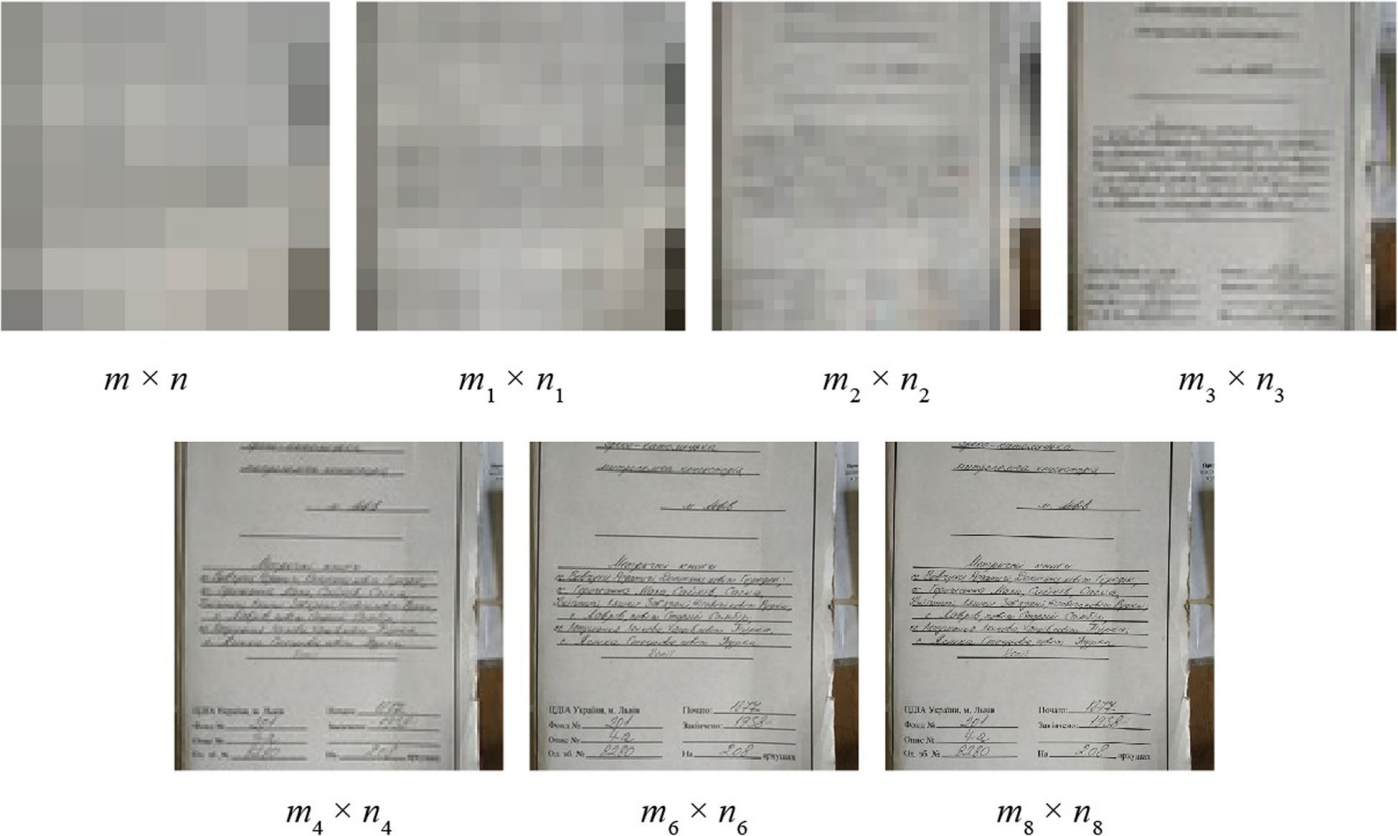
Images with different virtual pixel sizes

In Table 1; Figs. 2 and 3, the results of the calculations and experiments for the virtual sizes of the RCDOER-matrix with different physical pixel sizes are shown. In the calculations, the following equation was used:

12 $$m_\Sigma \times n_\Sigma =m\left( {1+{k_1}} \right) \times n\left( {1+{k_2}} \right):m_\Sigma =m\left( {1+{k_1}} \right);n_\Sigma =n\left( {1+{k_2}} \right)$$

13 $$K=\left( {1+{k_1}} \right) \times \left( {1+{k_2}} \right)$$

The calculation results are provided for case *k*_1_ = *k*_2_ = *k*. The magnitude of the multiplicity *k* is considered within the range of 1–8. The pixel dimensions of the RCDOER-matrix are as follows: 0.31, 2.0, and 4.0 MP.

The virtual pixel size increased 81-fold when the multiplicity of the pixel placement from 1 to 8 is changed. For a matrix with a physical size of 0.31 MP, the virtual size increases to 25.11 MP; for a 2 MP matrix, up to 162 MP; for a 4 MP matrix, up to 324 MP. Examples of images with the virtual pixel size of the equivalent matrix are shown in Fig. 3. Information about the parameters of the equipment that was used in the experiment is presented in Table 2. For modeling, a part of the image had a pixel size of 16 × 16.

**Table 1:** Physical and virtual pixel size of RCDOER-matrix

Pixel size of the RCDOER-matrix, *m* × *n* (MP)	Virtual pixel size of equivalent matrix, $m_\Sigma \times n_\Sigma$, at *k* (*k*_1_ = *k*_2_ = *k*) (MP)					
	1	2	3	4	6	8
0.31	1.24	2.79	4.96	7.75	15.19	25.11
2.00	8.00	18.00	32.00	50.00	98.00	162.00
4.00	16.00	36.00	64.00	100.00	196.00	324.00

**Table 2:** Hardware parameters

Model of hardware	Light sensitivity	Pixel size of thematrix	Pixel size of the image	Resolution
Canon Power Shot SX 420 IS Black	400 S_ISO_	20 MP	5152 × 3864	2 × 10^−6^*m*

In addition, a change in the mechanism is needed for forming and saving a frame with the observation results. Snapshot contains signals from pixels. For the *r*-th snapshot, the *x*_*r,i,j*_ signal comes from pixel located in the *i*-th row and *j*-th column of the RCDOER-matrix. The set of all values of the snapshot signals comprises a rectangular matrix of *m* × *n* in size. None of the snapshots contained duplicate pixels.

In this case, Eqs. (9) and (10) are considered as matrices. The elements of the matrix description of the frame are signals from the pixels of all images in the snapshots. The matrix description of the frame image contains matrix elements of the following types [14]:

14 $$ \mathrm{X}=\left[\begin{array}{*{20}c}{x}_{111} & \ldots &{x}_{{r_1}11} & \ldots &{x}_{\left({k}_1+1\right)1m} \\ {}\vdots & & \ddots & & \vdots\\ {}{x}_{K\left({k}_2+1\right)n1} & \ldots &{x}_{r_2\left({k}_2+1\right)n1} & \ldots &{x} {r_2\left({k}_2+1\right)n\left({k}_1+1\right)m}\end{array}\right]$$

For Fig. 1, the added snapshots are taken at the displacement along the *r*_1_ row and *r*_2_ column. In this case,

$$r={r_1}+{r_2};{r_1}=\left( {\overline {{1,{k_1}+1}} } \right);{r_2}=\left( {\overline {{1,{k_2}+1}} } \right)$$

Then, Eq. (10) can be rewritten in a matrix form:

15 $$X=\sum\limits_{{r=1}}^{{r=K}} {{X_r}}$$

The matrices **X** and **X**_r_ must have the same size horizontally and vertically. Only such a matrix can be added.

From Eq. (13), it follows that the values of $m_\Sigma$ and $n_\Sigma$ are determined by Eq. (12). The size of the matrix signals of each snapshot should be $m_\Sigma \times n_\Sigma$. To achieve this, zeros were added to the columns and rows. The rows and columns that were not used in this snapshot were given a value of zero. If ${k_1} \ne 0$, zero rows should appear, if ${k_2} \ne 0$ zero columns should appear. If ${k_1} \ne 0$ and ${k_2} \ne 0$, then zero rows and columns will appear.

The general rule for the sequence of recording signals from the frame pixels will be as follows [14]:

16 $$ j=\left\{ {{j_r}:{j_r} \in \left( {\overline {{1,K}} } \right),j \in \left( {\overline {{m \times n}} } \right),r=\left( {\overline {{1,K}} } \right),K=\left( {1+{k_1}} \right)\left( {1+{k_2}} \right)} \right\}$$

where *j*_*r*_ is the *i*-th pixel number in the *r*-th frame snapshot.

It is necessary to observe the sequence of pixels, which are recorded by both the number of elementary receivers and the number of snapshots. It is convenient to form a frame from snapshots in a matrix form. The structure of the frame memorization register is determined by dimension of the image memorization register, as well as the trajectory of the image movement during intra-matrix scanning. The simplest version of the trajectory is linear. In this case, the measured value *x*_*r,i,j*_ is written into each *ij*-cell of the register sequentially after each snapshot.

For observation devices with a conventional photosensitive matrix, the concepts of a frame and a snapshot coincide: $X={M_s}\left\{ {{x_i}} \right\}={M_f}\left\{ {{x_i}} \right\}$. The sets of *M*_*s*_ and *M*_*f*_ correspond to the number of bins *N* of the image. The number of bins *N* of image *X* is equal to the number of elementary receivers (*m* × *n*) of the matrix. The same number is for the bins per frame, i.e., $X=\left\{ {{x_i}} \right\}$:

17 $${X_s}={X_f}=\sum\limits_{{j=1}}^{{j=m}} {\sum\limits_{{i=1}}^{{i=n}} {{x_{i,j}};\sup \left\{ {i,j} \right\}} } =\left( {m \times n} \right)=N$$

When constructing an image using the RCDOER-matrix, a frame with *M*_*f*_ sampling elements is formed from *K* snapshots. Each snapshot had *N* bins. The design of the RCDOER-matrix and scanning system ensures that certain conditions are met.

18 $$\begin{array}{*{20}c} X=\left\{ {{X_r}\left\{ {{x_r},i} \right\}} \right\}=\sum\limits_{{r=1}}^{K} {{X_r}\left\{ {{x_r},i} \right\}} =\sum\limits_{{r=1}}^{{r=K}} {\sum\limits_{{j=1}}^{{j=m}} {\sum\limits_{{i=1}}^{{i=n}} {{x_{r,i,j}}} } } \hfill \\ \sup \left\{ {i,j} \right\}=N,\sup \left\{ r \right\}=K:\sup \left\{ {r,i,j} \right\}=N \times k={M_f} \hfill \\ {X_r} \cap {X_k}=\phi ;k,r=\overline {{1,k}} ;k \ne r \hfill \\\end{array}$$

At the physical level, Eq. (18) provides a digital representation of the environment. Two basic requirements are formalized. Equations (14), (15), (17), and (18) set the relationship between the number of snapshots in a frame. This is a condition for the completeness of documentation to all discrete elements of space Ω.

The fulfillment of the lower condition presupposes the absence of common signals from sampling elements of space Ω in the snapshots. This is the condition for the absence of intersections between images.

The size of the frame matrix horizontally (1 + *k*_1_)-fold and vertically (1 + *k*_2_)-fold is larger than the size of the photograph frame matrix. The matrix summation is applied only with matrices of the same size. Therefore, for ${k_1},{k_2} \ne 0$, zero rows and columns must be entered into the snapshot signal of the matrix. If ${k_1} \ne 0$, zero rows should appear, if ${k_2} \ne 0$, zero columns should appear. In terms of hardware, the matrix analogy simplifies the register distribution of signals from pixels and the reproduction of a frame image.

Hardware requirements need to be defined. Let us describe the theoretical justification for the time that is need for creating snapshots and frames. The exposure time of one snapshot is *t*_*e*_. Moving the matrix by one pixel (2, …, 8 μm) is *t*_*s*_. This did not exceed the exposure time. During this time, the p-n transitions of photosensitive receivers relax (10^−6^ s). The exposure time *t*_*f*_ of the frame is as follows:

19 $${t_f}=\left( {{k_1}+{k_2}+2} \right)\left( {{t_e}+{t_s}} \right)=K\left( {{t_e}+{t_s}} \right)$$

The standard average shutter speed at the lowest sensitivity level in the camera is 0.01 s. Then, up to 20 frames were formed in 1 s. With an average photosensitivity, this is already several tens of frames, according to Eq. (19). This frame rate will allow video recording.

Displacement of the image during formation of each frame is not a problem. In each camera there is a mechanism for compensating for image-shift. It provides image displacement in much the same way as RCDOER-matrix.

Physical limitations on the possibility of observation with RCDOER-matrix are: (1) the minimum possible or reasonable pixel density; and (2) precision regarding the realization of the image scanning trajectory.

We can use RCDOER-matrices in all technical devices designed to observe objects by its digital image. The advantages of using RCDOER-matrices as a receiver of radiation are as follows: (1) obtaining of a digital image with pixel size that is multiples of the pixel size of the matrix (all pixels of a digital image are result of physical measurement of the brightness of the conjugate area, and it is not the result of calculation); (2) simplification of the technology of manufacturing photosensitive matrices (this is important if you need to use multi-megapixel matrices); and (3) decrease of diffraction influence at elementary receiver of the matrix on measured values of brightness in neighboring pixels (this property is interesting when miniaturizing the size of photosensitive matrices, as well as when constructing images in the high frequency range).

For using RCDOER-matrices in real equipment it is necessary to solve the following issues: (1) to determine the rational level of density of elementary receivers in the photosensitive matrix [it corresponds of multiplicity of the arrangement of elementary receivers along the horizontal and vertical (Eqs. 11, 12, and 13), the limiting factor is the degree of dynamism of the object that is supposed to be observed with the equipment, as consequence, this is a limitation on the number of shots in a single frame (Eq. 10)]; (2) to set the trajectory of movement of optical image along the RCDOER-matrix [the reproduction of observation depends on accuracy of the trajectory (Eq. 18)]; (3) to place accurately the elementary receivers in RCDOER-matrix [it will provide adequate representation of the observed picture in its digital image and prevent intersections between images of individual snapshots (Eq. 18)]; and (4) to adapt the recording of signals from pixels of the frame to the conditions of using the RCDOER-matrix (Eq. 16).

## Conclusions

Observation devices with a matrix are used in various fields, including medicine. Sampling of the image into pixels will decrease spatial information of the object. This disadvantage can be avoided by using RCDOER-matrix. For example, with it we can get 324 megapixel images by using a 4 megapixel matrix. We do not need to increase the pixel size of the photosensitive matrix. It is achieved by inside matrix scanning of the optical image. The algorithms that were developed for the functioning of RCDOER-matrix have a declarative nature. The research results, formalize tasks that arise for creating prototypes of RCDOER-matrix. The conditions for hardware implementation are proposed.

The structure of the frame memorizing register is proposed. Also, it is determined by the dimension of the image memorizing register, as well as the trajectory of the image movement during intra-matrix scanning. The disadvantages of using RCDOER-matrices in observation devices are assessed. Thus, the results of the research carried out approximate the practical application of RCDOER-matrices for observation devices.

## Data Availability

Not applicable.

## Abbreviations

RCDOER: Regularly changing (controlled) density of elementary receivers
